# Incidence and risk factors associated with postoperative stroke in the elderly patients undergoing hip fracture surgery

**DOI:** 10.1186/s13018-020-01962-6

**Published:** 2020-09-18

**Authors:** Lili Yu, Yanbin Zhu, Wei Chen, Hui Bu, Yingze Zhang

**Affiliations:** 1 Department of Neurology, The 2nd Hospital of Hebei Medical University, Shijiazhuang, 050000 Hebei People’s Republic of China; 2 Key Laboratory of Neurology of Hebei Province, Shijiazhuang, 050000 Hebei People’s Republic of China; 3 Department of Orthopaedic Surgery, The 3rd Hospital of Hebei Medical University, Shijiazhuang, 050051 Hebei People’s Republic of China; 4 Key Laboratory of Biomechanics of Hebei Province, Shijiazhuang, 050051 Hebei People’s Republic of China; 5 Chinese Academy of Engineering, Beijing, 100088 People’s Republic of China

**Keywords:** Epidemiology, Hip fracture, Multivariate analyses, Risk factors, Stroke

## Abstract

**Objectives:**

Stroke is one of the rare but devastating complications after hip fracture in the elderly. By far, there is still scarce data on postoperative stroke in elderly patients with hip fractures.

**Methods:**

This was a retrospective study of prospectively collected data. Between October 2014 to December 2018, patients aged above 65 years who underwent operative treatment for hip fractures were included. Inpatient medical surveillance and scheduled telephone follow-up at 1, 3, 6, and 12 months after operation was conducted to identify who developed an incident stroke. Variables of interests were extracted from patients’ inpatient medical records. Univariate analysis and multivariate logistic regression analysis were used to identify the independent risk factors associated with stroke.

**Results:**

During the study period, a total of 3743 patients were included, among whom 56 were found to have a stroke after operation, representing an incidence of 1.5% (95% CI, 1.1 to 1.9%). The multivariate analyses showed that advanced age (1-year increment; OR, 1.32; 95% CI, 1.08 to 1.48), history of previous stroke (OR, 4.79; 95% CI, 1.86 to 6.56), ASA III and above (OR, 2.62; 95% CI, 1.27 to 3.68), long-term use of aspirin (OR, 3.63; 95% CI, 1.41 to 4.78), and elevated RDW level (each increment of 1%, OR, 1.21; 95% CI, 1.02 to 1.36) were independently associated with postoperative stroke.

**Conclusions:**

Although most are not modifiable, these risk factors help in counseling patients regarding the risk of postoperative stroke, individual risk stratification, and targeted optimization of medical conditions and should be firmly kept in treating surgeon’s mind.

## Introduction

With the aggravation of aging in the worldwide, osteoporotic fracture has become one of the major threats to health-care systems [1, 2]. As a typical osteoporotic fracture, hip fracture is anticipated to increase to 4.5 to 6.3 million by 2050 [3], with Asian being the mostly affected population. A large epidemiological survey in China suggested hip fracture is the second largest type of fracture in the elderly population, only following the distal radius fracture [4]. Notable as the “last fracture” in one’s lifetime, hip fracture seriously affects the limb function and quality of life and substantially increases the social and family burden [5]. The complications after hip fractures have always been a focus subject, particularly the cardia-cerebrovascular disease including postoperative stroke [6]. Indeed, there is a large proportion of overlapping factors between hip fracture and stroke, such as advanced age, comorbidity, impaired vision, weakened muscle strength, cognitive impairment, and susceptibility to falls, so the incidence of stroke after hip fracture is increased by several folds [7–9]. In addition, during acute therapy in patients with hip fracture, traumatic stress, perioperative use of medications, surgical intervention, iatrogenic trauma, and postoperative pathophysiological changes also increased the risk of stroke [6, 10].

Previous studies have made various attempts to investigate the incidence and risk factors for postoperative delirium after hip fracture surgeries [11–14], or incident hip fracture after the stroke [8, 9, 15]. However, there is still a lack of epidemiologic data on postoperative stroke following surgery of hip fractures. By far as we know, there has been no study specifically addressing the incidence and risk factors associated with postoperative stroke following hip fracture surgeries. By comparison, most of the studies focused on surgical methods (hip arthroplasties or fracture repair) [16, 17], other bone fracture locations [18], or the crude impact of hip fracture on stroke [6, 19]. As such, the extrapolation of these results to studies of hip fractures might be inappropriate.

In this study, we used the prospectively collected data in university-affiliated orthopedics hospital, with aims to investigate the incidence rate of postoperative stroke after hip fracture surgeries and identify some independent risk factors associated with occurrence of stroke.

## Materials and methods

All methods used in this study were performed in accordance with the STROCSS (Strengthening the Reporting of Cohort Studies in Surgery) guidelines.

Data used in this study were extracted from the database of Surgical Site Infection in Orthopaedic Surgery (SSIOS). The SSIOS database is a prospectively maintained and updated database of all the data on hospitalized patients undergoing orthopedic surgeries, beginning from 1 October 2014 and updated yearly. The initial aim of SSIOS was to evaluate the surgical site infection (SSI) after orthopedic surgery using the method of medical surveillance during hospitalization stay and scheduled telephone visits after discharge. Before the SSIOS commenced, it was approved by the ethics committee of the 3rd Hospital of Hebei Medical University (No. 2014-015-1). All the participants have provided their written informed consent.

In this study, patients with hip fractures surgically treated in our hospital from October 2014 to December 2018 were included. Inclusion criteria were patients aged 65 years or older, definite diagnosis of hip fracture (femoral neck fracture or intertrochanteric fracture) caused by low-energy trauma (fall from standing height), surgical treatment (hip replacement or osteosynthesis), complete data available in medical records, and follow-up period for at least 12 months. Exclusion criteria were fractures caused by high-energy trauma, combined fractures in other locations, conservative treatment, patients with incomplete medical records, loss to follow-up, or death during follow-up period.

### Definition and diagnosis of stroke

Stroke was defined as acute onset of a neurologic deficit that corresponded to an arterial vascular territory of the cerebral hemispheres, brainstem, or cerebellum. Stroke was classified as hemorrhagic and ischemic stroke (cerebral infarction and transient ischemic attack (TIA)). Stroke was diagnosed based on patients’ consciousness, limb performance, head CT, and MRI performance.

### Data extraction

Patient data were extracted from electronic medical records. Baseline characteristics included residence (urban or rural), age, gender, body mass index (BMI), cigarette smoking, alcohol consumption, and co-morbidities (hypertension, diabetes, rheumatoid arthritis, history of ischemic heart disease, previous any fracture, previous history of stroke, and long-term use of aspirin (> 6 months before the hip fracture occurrence)). Fracture and surgery-related data included preoperative interval (between fracture occurrence and operation), the type of fracture (femoral neck or intertrochanteric), injury mechanism (high or low-energy injury), operative duration, blood loss, anesthesia type, and American Society of Anesthesiologists (ASA) classification, as well as laboratory indexes such as total protein (TP), albumin (ALB), alanine transaminase (ALT), aspartate transaminase (AST), preoperative red blood cell (RBC), white blood cell (WBC), hemoglobin (HGB), hematocrit (HCT), platelet (PLT), red blood cell distribution width (RDW), serum total cholesterol (TC), triglyceride (TG), low-density lipoprotein (LDL-C), high-density lipoprotein (HDL-C), and very low-density lipoprotein (VLDL). The BMI (kg/m^2^) was subgrouped using the criteria recommended by the Chinese working group on obesity: normal (18.5–23.9), underweight (< 18.5), overweight (24.0–27.9), and obesity (≥ 28.0). Low-energy injury was defined as an injury caused by a fall from a standing height, and others such as fall from a height, motor accidents were deemed as high-energy injury.

### Statistical analysis

The categorical data were expressed as frequency (*n*) and percentage (%) and were evaluated by Chi-square test or Fisher’s exact test. Continuous variables were expressed by mean and standard deviation (SD) and the difference was evaluated by Student *t* test or Mann-Whitney *U* test, as appropriate. A multivariate logistics regression analysis was used to identify the independent risk factors associated with occurrence of stroke, using the stepwise backward elimination method. The variables were remained in the final model when *p* value less than 0.10, and the correlation strength was indicated by odd ratio (OR) and 95% confidence interval (95% CI). The significance level was set as *p* < 0.05. Hosmer-Lemeshow (H-L) test was used to evaluate the fitting degree of the final logistics regression model, and *p* > 0.05 represented the acceptable result. All tests were performed using SPSS21.0 (IBM, Armonk, NY, USA).

## Results

### The general information

Totally, 3743 patients were deemed as eligible and included, including 1592 males and 2151 females. The average age was 78.2 years, ranging from 65 to 104 years. During the hospitalization and follow-up period, 56 patients had a stroke, indicating an accumulative incidence rate of 1.5% (56/3743; 95% CI, 1.1 to 1.9%).

Univariate analysis showed that the age of patients with stroke was significantly higher than that of the non-stroke group (80.3 vs 78.1, *p* = 0.001). The incidence rate of stroke was higher among patients undergoing osteosynthesis than those undergoing THA (1.7% vs 0.9%), although not significantly different (*p* = 0.103). Patients with a stroke had a comparable preoperative hospitalization stay for the hip fracture surgery as that of non-stroke patients (4.6 vs 4.3 days, *p* = 0.278). There were no significant differences between both groups in BMI, smoking status, surgical methods, ASA classification, and others between the two groups (*p* > 0.05) (Table 1) either.

**Table 1:** Association between potential risk factors and stroke after hip fracture surgeries

Variables	Number (%) of stroke (*n* = 56)	Number (%) of non-stroke (*n* = 3687)	***P***
**Gender (male)**	19 (33.9)	1573 (42.7)	0.189
**Age (years)**	80.3 ± 8.5	78.1 ± 11.4	0.001
**Living place**			0.482
Rural	29 (51.8)	1735 (47.1)	
Urban	27 (48.2)	1952 (52.9)	
**BMI (kg/m**^**2**^**)**	24.7 ± 5.6	25.4 ± 3.9	0.672
18.5–23.9	24 (42.9)	1368 (37.1)	0.221
< 18.5	5 (8.9)	198 (5.4)	
24.0–27.9	17 (30.4)	1595 (43.3)	
≥ 28.0	10 (17.9)	526 (14.3)	
**Diabetes mellitus**	11 (19.6)	538 (14.6)	0.289
**Hypertension**	19 (33.9)	895 (24.3)	0.095
**Chronic heart disease**	7 (12.5)	362 (9.8)	0.504
**Past stroke**	11 (19.6)	287 (7.8)	0.001
**Chronic use of aspirin**	10 (17.9)	325 (8.8)	0.019
**Injury mechanism (low-energy)**	53 (94.6)	3521 (95.5)	0.760
**Preoperative stay**	4.6 ± 3.2	4.3 ± 2.6	0.278
**Total hospital stay**	14.4 ± 7.4	15.1 ± 7.9	0.443
**Intraoperative bleeding**	286.6 ± 304.2	278 ± 187.1	0.643
**Surgical duration**	106.5 ± 47.0	97.8 ± 51.4	0.074
**Cigarette smoking**	9 (16.1)	774 (21.0)	0.369
**Alcohol consumption**	13 (23.2)	1402 (38.0)	0.023
**Fracture type**			0.149
**Femoral neck**	25 (44.6)	2005 (54.3)	
**Intertrochanteric**	31 (55.4)	1686 (45.7)	
**Procedure**			0.103
Osteosynthesis	47 (83.9)	2742 (74.4)	
Arthroplasty	9 (16.1)	945 (25.6)	
**Anesthesia**			0.145
Local	2 (3.6)	134 (3.6)	
Spinal	23 (41.1)	1989 (53.9)	
General	31 (55.4)	1564 (42.4)	
**ASA class**			0.007
I-II	31 (55.4)	2642 (71.7)	
III or above	25 (44.6)	1045 (28.3)	
**TP (g/L)**	54.1 ± 7.9	59.2 ± 7.2	0.005
**ALB (g/L)**	28.4 ± 4.5	33.6 ± 5.5	<0.001
**ALT (U/L)**	25.3 ± 19.4	24.5 ± 23.7	0.579
**AST (U/L)**	26.7 ± 16.4	26.2 ± 23.8	0.752
**FBG** (mmol/L)	5.1 ± 2.2	5.0 ± 1.9	0.896
**TC** (mmol/L)	3.8 ± 0.9	3.5 ± 0.7	0.103
**TG (**mmol/L**)**	1.3 ± 0.6	1.1 ± 0.7	0.164
**LDL-C (**mmol/L**)**	2.2 ± 0.6	2.4 ± 0.7	0.489
**HDL-C (**mmol/L**)**	0.9 ± 0.4	1.2 ± 0.3	0.016
**VLDL (**mmol/L**)**	0.6 ± 0.3	0.5 ± 0.3	0.133
**WBC (10**^**9**^**/L)**	9.0 ± 3.1	8.7 ± 3.3	0.673
**NEUT (10**^**9**^**/L)**	6.9 ± 2.9	6.9 ± 2.8	0.907
**LYM (10**^**9**^**/L)**	1.2 ± 0.6	1.1 ± 0.6	0.918
**RBC (< lower limit)**	26 (46.4)	2078 (56.4)	0.137
**HGB (< lower limit)**	19 (33.9)	858 (23.3)	0.062
**HCT (< lower limit)**	4 (7.1)	97 (2.6)	0.039
**PLT (< 100 × 10**^**9**^**/L)**	4 (7.1)	127 (3.4)	0.135
**PDW (%)**	15.7 ± 3.4	15.1 ± 2.4	0.176
**RDW (%)**	15.8 ± 1.5	14.1 ± 1.8	0.014

*BMI* Body mass index, *ASA* American Society of Anesthesiologists, *RBC* Red blood cell; reference range: female, 3.5–5.0/10^12^/L; males, 4.0–5.5/10^12^/L. *HGB* Hemoglobin; reference range: females, 110–150 g/L; males, 120–160 g/L. *FBG* Fasting blood glucose, *ALT* Alkaline phosphatase, *HCT* Hematocrit; 40–50%. *WBC* White blood cell, *NEUT* Neutrophile, *LYM* Lymphocyte, *PLT* Platelet; 100–300 × 10^9^/L. *TP* Total protein, *ALB* Albumin, *GLOB* Globulin, *RDW* Red cell distribution width, *PDW* Platelet distribution width, *TC* Total cholesterol, *TG* Triglyceride, *LDL-C* Low-density lipoprotein, *HDL-C* High-density lipoprotein, *VLDL* Very low-density lipoprotein

In the multivariate model, five independent risk factors were identified to be associated with postoperative stroke after hip fracture, including advanced age in 1-year increment (OR, 1.32; 95% CI, 1.08 to 1.48), history of previous stroke (OR, 4.79; 95% CI, 1.86 to 6.56), ASA III and above (OR, 2.62; 95% CI, 1.27 to 3.68), long-term use of aspirin (OR, 3.63; 95% CI, 1.41 to 4.78), and elevated RDW level (each increment of 1%, OR, 1.21; 95% CI, 1.02 to 1.36) (Table 2). As for other variables, we did not find their significant effect on the multivariate model. The H-L analysis demonstrated the good fitness of the final model (*X*^2^ = 3.186, *p* = 0.623; Nagelkerke *R*^2^ = 0.402).

**Table 2:** Multivariate analyses of risk factors associated with stroke after hip fracture surgeries

Variable	OR and 95% CI	*p* value
Age (increase of each year)	1.32 (1.08 to 1.48)	0.002
Previous stroke	4.79 (1.86 to 6.56)	< 0.001
ASA III and above	2.62 (1.27 to 3.68)	0.006
Long-term use of aspirin	3.63 (1.41 to 4.78)	0.011
Elevated RDW level (each increment of 1%)	1.21 (1.02 to 1.36)	0.027

*ASA* American Society of Anesthesiologists, *OR* Odd ratio, *CI* Confidence interval, *RDW* Red cell distribution width

## Discussion

Stroke is a serious complication after hip fractures in the elderly and can lead to increased morbidity, mortality, and medical costs. Surgical treatment of acute hip fractures may increase the risk of stroke for a variety of reasons. In this study, we found the incidence rate of stroke was 1.5% following hip fracture surgeries. Several risk factors associated with the occurrence of postoperative stroke were identified, including advanced age, history of stroke, higher ASA grade, long-term use of aspirin, and elevated preoperative RDW.

Due to the differences in study design, data resource, patient characteristics, and the follow-up period, the incidence rates were reported to be greatly varied in literature. The National Trauma Data Bank of the USA included 37,584 elderly patients with hip fractures between 2011 and 2012 and identified 162 patients who had a stroke during hospitalization stay, representing an incidence rate of 0.4% [20]. In another study of elderly veterans, the incidence rate of stroke was found to be 1% during hospitalization; however, the study time of 1982 to 1993 and specifying at a special occupational group might compromise generalizability of the results [21]. The relatively lower incidence rate of stroke in both studies was largely attributable to the shorter follow-up period that was only in hospitalization stay. Recent studies have reported higher incidence rates of stroke after hip fracture, reaching 4% at the follow-up of 1 year [17, 19], about 2.5-fold higher than ours. Despite with various results reflecting the heterogeneity among studies, these studies did provide useful information about stroke after surgery of hip fractures, also aided in understanding of the higher rate of 1-year mortality or independence loss [22].

Advanced age was identified as an independent risk factor for stroke in our study, consistent with that of previous studies [18, 23]. But this relationship does not exclude the confounding effects of factors other than those included in this study, such as increased prevalence of osteoporosis, cognitive impairment, or other medical conditions in elderly patients. It should also be noted that old age is a primary overlapping factor among hip fracture and stroke patients, and therefore, the role of advanced age as a risk factor in causing stroke might not be pure and should be viewed in context of interactions between co-morbidities, impaired organ functions, and age [6], although some of them were adjusted in our multivariate model. Particular attention should be paid to patients with a history of previous strokes, in whom the risk of a recurrent stroke was increased by 13 times [24]. Therefore, patients with a previous history of stroke should be classified as an extremely high-risk group, which deserved more attention and optimization of medical conditions. It is suggested that for patients with recent stroke or transient ischemic attack, the surgical operation should be delayed for at least 9 months, using local anesthesia instead of general anesthesia, to avoid occurrence of perioperative hypotension and the recurrence of perioperative stroke [25].

ASA level is an important indicator for co-morbidities and anesthesia risk index. This study found a significant positive correlation between higher ASA level and postoperative stroke occurrence. Similar as our findings, previous studies found a positive correlation between the risk of stroke and the number of chronic diseases [6, 10]. Therefore, it is of significance to optimize surgical conditions and control parameters for chronic diseases during hospitalization to reduce the incidence of stroke.

The use of anticoagulants or anti-platelet drugs are increasingly prevalent, due to their positive effects on reduction of thromboembolic events [26], but their risk in stroke has been always a controversy. Samuel et al. [20] demonstrated the increased 65% risk of stroke after long-term use of anticoagulants in elderly hip fracture patients, independent of other variables. Popa et al. [17] found aspirin use was not a significant predictor of postoperative stroke after adjustment for other variables and only considered it as a marker of increased cardiovascular risk. In this study, although we observed a significant association between aspirin and the stroke, this correlation should be treated cautiously because it might also be the reflection of previous history of stroke or other thromboembolic events. The role of long-term use of aspirin in cerebrovascular event requires prospective, larger sample-size cohort studies to confirm.

The association between laboratory RDW values and stroke has been demonstrated in previous studies [27–29], but this is the first report specifying hip fractures. Lappegard et al. [27] evaluated 25,992 participants in a prospective cohort study, of whom 1152 participants had their first stroke at a median follow-up of 15.8 years. After adjusting for multiple variables, the authors found every 1% increase in RDW was associated with increased 13% risk of stroke, and participants with RDW values in the highest quartile had a 37% higher risk of stroke than those with RDW values in the lowest quartile. Moreover, Kim et al. [30] confirmed that in stroke patients, every 1% increase in RDW value was associated with the increased 1.3-fold mortality rate, and furthermore this effect persisted throughout the follow-up period (> 1 year). Therefore, close monitoring of this biomarker index is of great importance in evaluating the prognosis, not only occurrence of but even the death.

Despite a large sample and a relatively long follow-up period, this study suffered from several limitations. Firstly, this study was still unable to evaluate the effects of some infrequent medical conditions, such as renal insufficiency and chronic use of glucocorticoid. Secondly, we were unable to investigate the relationship between some complications and occurrence of stroke, including surgical site infection, deep venous thrombosis, and others. Thirdly, due to using the telephone visit, we could not know the accuracy in diagnosis of stroke and its subclassifications. Fourthly, the results of this study might not be extrapolated to others, due to the difference in race, ethnic, or the specific operative technique.

In summary, we found the incidence rate of stroke after surgery of hip fracture was 1.5% at the follow-up above 1 year and identified 5 independently associated risk factors: advanced age, history of previous stroke, ASA III and above, long-term use of aspirin, and elevated RDW level. Although most of them are not modifiable, they could provide data for construction of predictive models for stroke occurrence and aid in counseling patients regarding the stroke risk after operation.

## Data Availability

All the data will be available upon motivated request to the corresponding author of the present paper.

## Abbreviations

SSI: Surgical site infection
BMI: Body mass index
EMR: Electronic medical records
ASA: American society of anesthesiologists
DM: Diabetes mellitus
SD: Standard deviation
OR: Odd ratio
RBC: Red blood cell
HGB: Hemoglobin
ALB: Albumin
FBG: Fasting blood glucose
HCT: Hematocrit
WBC: White blood cell
NEUT: Neutrophile
LYM: Lymphocyte
PLT: Platelet
TP: Total protein
GLOB: Globulin
PDW: Platelet distribution width
SSIOS: Surgical Site infection in orthopaedic surgery
TIA: Transient ischemic attack
ALT: Alanine transaminase
AST: Alanine transaminase
RDW: Red blood cell distribution width
TC: Total cholesterol
TG: Triglyceride
LDL-C: Low-density lipoprotein
HDL-C: High-density lipoprotein
VLDL: Very low-density lipoprotein
H-L test: Hosmer-lemeshow test
THA: Total hip arthroplasty
